# Frailty and risk of gastrointestinal bleeding: a prospective cohort study based on UK biobank

**DOI:** 10.3389/fpubh.2025.1625869

**Published:** 2025-07-04

**Authors:** Chenao Zhang, Qiming Huang, Xingyu Liu, Jiren Wang, Junyan Wang, Jian Song, Rong Song, Hong Su, Qiao Mei

**Affiliations:** ^1^Department of Gastroenterology, The First Affiliated Hospital of Anhui Medical University, Hefei, China; ^2^Department of Epidemiology and Health Statistics, School of Public Health, Anhui Medical University, Hefei, Anhui Province, China

**Keywords:** frailty, gastrointestinal bleeding, cohort study, Cox proportional hazard model, UK biobank

## Abstract

**Introduction:**

Frailty has been associated with various diseases. However, its impact on gastrointestinal bleeding (GIB) remains largely unexplored. This study investigates the relationship between frailty and the incidence of gastrointestinal bleeding events.

**Methods:**

A total of 352,060 participants from the UK Biobank with no history of gastrointestinal bleeding were included. Baseline frailty status was assessed using the Fried phenotype and categorized as non-frail, pre-frail, or frail. The primary outcome was gastrointestinal bleeding, identified through hospitalization records and death registries. Cox proportional hazard models were used to evaluate the association between frailty and gastrointestinal bleeding incidence.

**Results:**

Among the 352,060 participants (mean age 56.1 years), 3.6% (*N* = 12,747) were classified as frail, and 43.6% (*N* = 153,424) as pre-frail at baseline. Over a median follow-up of 14.7 years, 20,105 gastrointestinal bleeding events were recorded. Compared to non-frail individuals, frail (HR = 1.53, 95% CI: 1.44–1.62) and pre-frail (HR = 1.15, 95% CI: 1.11–1.18) individuals exhibited a significantly higher risk of gastrointestinal bleeding after multivariate adjustment (*P* for trend < 0.001). Subgroup and sensitivity analyses remained consistent findings.

**Conclusion:**

Frailty significantly elevates the risk of gastrointestinal bleeding. Early identification and targeted multidimensional interventions addressing frailty may reduce gastrointestinal bleeding events and improve patient prognosis.

## 1 Introduction

Gastrointestinal bleeding (GIB) is a prevalent and severe gastrointestinal emergency worldwide that has been the focus of extensive research. Recent epidemiological data indicate that, in the United States alone, over 500,000 hospital admissions occur annually due to GIB, resulting in direct medical costs of approximately $5 billion and nearly 11,000 deaths. Furthermore, GIB contributes significantly to healthcare utilization, accounting for more than 2 million inpatient days annually, highlighting its substantial clinical and economic burden (1). Clinical manifestations of GIB range from mild symptoms to life-threatening conditions, frequently necessitating timely interventions including endoscopic procedures, blood transfusions, and admission to intensive care units (2). Therefore, identifying risk factors is essential for reducing the incidence of GIB and optimizing clinical management and resource allocation. GIB risk is influenced by numerous factors, including demographic characteristics (e.g., advanced age), lifestyle habits (e.g., alcohol consumption, smoking), infectious agents (e.g., *Helicobacter pylori*), medication use (e.g., nonsteroidal anti-inflammatory drugs [NSAIDs], glucocorticoids), and comorbidities (e.g., diabetes mellitus, chronic kidney disease, malignant neoplasms) (2–5). Nonetheless, the limited predictive accuracy of current risk models suggests the existence of unidentified biological factors influencing susceptibility to GIB (6).

Frailty is a biomedical syndrome characterized by a cumulative decline in multisystem physiological functions, predominantly affecting older adults (7–9). It has been increasingly recognized as a reliable marker for predicting adverse health outcomes. Frail individuals exhibit heightened susceptibility to various adverse conditions, consequently leading to increased healthcare costs and utilization of medical resources (10). The Frailty Phenotype (FP) is quantitatively assessed using five criteria established by the Fried model: weight loss, exhaustion, low physical activity, slow walking speed, and low grip strength (11). Growing evidence indicates a strong association between frailty and several pathophysiological alterations, including chronic inflammatory activation, autonomic dysfunction, and dysregulation of coagulation and fibrinolytic processes (12, 13). Numerous studies have reported associations between frailty and the development of diverse diseases, such as cardiovascular disease, chronic liver disease, and Alzheimer’s disease (14–16). Thus, it is plausible that frailty contributes to an increased risk of gastrointestinal bleeding. However, most existing studies have primarily focused on the influence of frailty on clinical outcomes and prognosis in patients already diagnosed with gastrointestinal bleeding (17, 18). Prospective, large-scale, population-based studies exploring whether frailty independently increases GIB risk remain limited.

To address the identified research gaps, this study utilizes long-term follow-up data from the UK Biobank cohort and employs a standardized frailty assessment system to investigate the association between frailty and the incidence of GIB events. The study aims to identify novel pathways for the early detection and preventive intervention of high-risk populations.

## 2 Materials and methods

### 2.1 Study design and population

The UK Biobank (UKB) is a large-scale, prospective cohort study. Between 2006 and 2010, over 500,000 participants aged 40 to 69 years were recruited, and extensive phenotypic and genotypic data were collected, including longitudinal follow-up data from touchscreen questionnaires, brief interviews, physical measurements, and biological samples. The study aimed to conduct a comprehensive investigation into the genetic and non-genetic determinants of midlife and age-related diseases (19). The study received ethical approval from the UK North West Multicentre Research Ethics Committee (Reference 21/NW/0157), and all participants provided informed consent upon enrolment.

Of the 502,356 participants initially enrolled, individuals who withdrew from the study (*N* = 223), those with missing frailty-related variables (*N* = 133,960), and those with incomplete covariate data (*N* = 6,863) were excluded. For the analysis of GIB events, participants with a history of gastrointestinal bleeding at baseline (*N* = 9,250) were also excluded (Supplementary Figure S1). The follow-up period concluded on October 25, 2023. This study followed the Strengthening the Reporting of Observational Studies in Epidemiology (STROBE) guidelines for cohort studies (20).

### 2.2 Assessment of frailty

Frailty assessment was based on five distinct dimensions: weight loss, exhaustion, physical activity, walking speed, and grip strength (11). ① *Weight loss:* Participants were asked, “*Compared to one year ago, has your weight changed?*” Those who answered “*Yes, lost weight*” were classified as experiencing weight loss, whereas responses of “*No, weigh about the same*” or “*Yes, gained weight*” were categorized as no weight loss. ② *Exhaustion:* Participants were asked, “*Over the past two weeks, how often have you felt tired or had little energy?*” Participants who selected “*More than half the days*” or “*Nearly every day*” were classified as experiencing exhaustion, whereas those who chose “*Not at all*” or “*Several days*” were not. ③ *Physical activity:* Total weekly metabolic equivalent of task (MET) minutes were calculated for each participant using the International Physical Activity Questionnaire (IPAQ). These values were adjusted for age and sex, and individuals with MET minutes in the lowest 20% were classified as having low physical activity. ④ *Walking speed:* Participants were asked, “*How would you describe your usual walking pace?*” Those who responded “*Slow pace*” were categorized as having low walking speed. ⑤ Grip strength: Handgrip strength was measured using a Jamar J00105 hydraulic manual dynamometer. The higher value from measurements of right and left-hand grip strength was selected, and low grip strength was defined using threshold values adjusted for gender and body mass index (BMI; Supplementary Table S1 for details).

FP scores ranged from 0 to 5, with higher scores indicating greater frailty. Participants were categorized as non-frail (FP score = 0), pre-frail (FP score 1 or 2), and frail (FP score ≥ 3).

### 2.3 Assessment of outcome

The primary outcome of this study was the incidence of gastrointestinal bleeding. Outcome determination adhered to standardized procedures from previous UK Biobank studies (21), integrating three data sources: (1) hospital inpatient records, based on ICD-9 and ICD-10 diagnostic codes; (2) death registration records, where ICD-10 codes identified GIB as the primary cause of death; and (3) self-reported data from participants at baseline. Follow-up time was calculated from the baseline to the occurrence of GIB diagnosis, death, loss to follow-up, or censoring, whichever came first. To ensure methodological reproducibility, the criteria for defining baseline and outcome events are provided in Supplementary Table S2.

### 2.4 Assessment of covariates

Covariates were selected based on prior epidemiological evidence (2–5), considering both their biological plausibility as potential confounders and data availability. Four categories of covariates were included in the final analysis: (1) Demographic characteristics, including age (continuous), sex and ethnicity; (2) Socioeconomic and lifestyle factors, including the Townsend Deprivation Index (TDI), education level, smoking status, alcohol intake frequency, and body mass index (BMI); (3) Medication use, assessed through a structured questionnaire and prescription records, covering the use of NSAIDs, other high-bleeding-risk medications and proton pump inhibitors (PPIs; all dichotomized as Yes/No); and (4) Clinical comorbidities, identified using International Classification of Diseases (ICD) codes in combination with self-reported data (dichotomous variables: Yes/No), including hypertension, diabetes, cardiovascular disease, chronic kidney disease, high-risk digestive disease, and cancer. Detailed descriptions of each covariate are provided in Supplementary Table S3.

### 2.5 Statistical analysis

For baseline data, continuous variables were described using mean ± standard deviation or median (interquartile range, IQR), while categorical variables were presented as percentages. Groups were stratified by frailty phenotype, and demographic characteristics, lifestyle factors, and comorbidity distributions were compared between groups. One-way analysis of variance (ANOVA) or the Kruskal-Wallis test was used for continuous variables, and the chi-squared test was applied for categorical variables.

Cox proportional hazard model was conducted to examine the association between frailty and incident GIB. In addition to univariable analysis, three multivariable adjustment models were constructed: (1) Model 1: adjusted for sex and age only; (2) Model 2: added demographic characteristics and lifestyle factors to Model 1, including race, Townsend Deprivation Index, education level, smoking status, and alcohol intake frequency; (3) Model 3: further adjusted for comorbidities (hypertension, diabetes, cardiovascular disease, chronic kidney disease, high-risk digestive disease, cancer) and medication use (NSAIDs, other high-bleeding-risk medications, PPIs). Hazard ratios (HRs) and their 95% confidence intervals (95% CIs) were reported, and the proportional hazards assumption was tested using the Schoenfeld residuals test. Kaplan–Meier curves for bleeding events were plotted for the different frailty phenotype groups, and differences between groups were compared using the log-rank test. Restricted cubic splines (RCS) were applied to explore the nonlinear relationship between continuous frailty phenotype scores and the risk of GIB, and the likelihood ratio test was performed to confirm the dose–response trend.

On sensitivity analyses: ① Subgroup analyses were performed by 12 dimensions such as age (<65/≥65 years), sex, and Townsend deprivation index, and interactions were assessed by likelihood ratio tests. ② Patients with GIB within 2 years of follow-up (*N* = 2,611) were excluded to rule out reverse causality; ③ the Fine & Gray method was used to correct for competing risks of all-cause mortality; ④ We also examined the correlations between the five frailty components and the risk of GIB.

All statistical analyses were performed by R version 4.4.1 (The R Project) between February and March 2025. A two-sided *p*-value <0.05 was considered statistically significant.

## 3 Results

### 3.1 Baseline characteristics

Table 1 presents the baseline characteristics of the study cohort, which included 352,060 participants without a history of GIB (mean age 56.1 ± 8.08 years; 47.7% male). Using the modified Fried frailty phenotype criteria, participants were classified as frail (3.6%, *N* = 12,747), pre-frail (43.6%, *N* = 153,424), or non-frail (52.8%, *N* = 185,889). Comparative analysis revealed significant demographic and clinical differences across frailty categories. Both the frail and pre-frail groups exhibited older mean ages, higher proportions of female participants, elevated body mass index, higher socioeconomic deprivation, and lower educational attainment compared to the non-frail group. These groups also demonstrated higher comorbidity burdens, including diabetes mellitus, cardiovascular disease, cancer, chronic kidney disease, and high-risk digestive disease, along with increased medication use.

**Table 1:** Baseline characteristics by frailty category.

Characteristics	Overall *(N = 352,060)*	Non-frail *(N = 185,889)*	Pre-frail *(N = 153,424)*	Frail *(N = 12,747)*	*p*-value
Sex					**<0.001**
Male	168,050 (47.7%)	91,617 (49.3%)	71,360 (46.5%)	5,073 (39.8%)	
Age^a^	56.1 (8.08)	56.0 (8.10)	56.1 (8.07)	57.5 (7.68)	**<0.001**
Ethnicity					**<0.001**
White	321,301 (91.3%)	171,086 (92.0%)	139,005 (90.6%)	11,210 (87.9%)	
TDI^b^	−2.28 [−3.71;0.22]	−2.43 [−3.80;-0.13]	−2.16 [−3.65;0.46]	−0.81 [−3.00;2.38]	**<0.001**
BMI^b^	26.6 [24.0;29.6]	25.9 [23.6;28.6]	27.3 [24.6;30.5]	30.2 [26.5;34.7]	**<0.001**
Education level					**<0.001**
College or University	128,468 (36.5%)	71,729 (38.6%)	53,699 (35.0%)	3,040 (23.8%)	
Smoking status					**<0.001**
Never	194,032 (55.1%)	104,726 (56.3%)	83,274 (54.3%)	6,032 (47.3%)	
Previous	123,174 (35.0%)	64,844 (34.9%)	53,728 (35.0%)	4,602 (36.1%)	
Current	34,854 (9.90%)	16,319 (8.78%)	16,422 (10.7%)	2,113 (16.6%)	
Alcohol intake frequency					**<0.001**
Daily or most daily	76,562 (21.7%)	44,331 (23.8%)	30,538 (19.9%)	1,693 (13.3%)	
Three or four times a week	86,062 (24.4%)	50,017 (26.9%)	34,389 (22.4%)	1,656 (13.0%)	
Once or twice a week	90,979 (25.8%)	48,075 (25.9%)	40,077 (26.1%)	2,827 (22.2%)	
One to three times a month	38,385 (10.9%)	18,506 (9.96%)	18,260 (11.9%)	1,619 (12.7%)	
Never or special occasions only	60,072 (17.1%)	24,960 (13.4%)	30,160 (19.7%)	4,952 (38.8%)	
Diabetes	16,864 (4.79%)	4,959 (2.67%)	9,674 (6.31%)	2,231 (17.5%)	**<0.001**
Hypertension	88,108 (25.0%)	38,969 (21.0%)	43,365 (28.3%)	5,774 (45.3%)	**<0.001**
Cardiovascular disease	21,974 (6.24%)	8,845 (4.76%)	10,842 (7.07%)	2,287 (17.9%)	**<0.001**
Chronic kidney failure	821 (0.23%)	284 (0.15%)	414 (0.27%)	123 (0.96%)	**<0.001**
Cancer	30,143 (8.56%)	14,992 (8.07%)	13,541 (8.83%)	1,610 (12.6%)	**<0.001**
High-risk digestive disease	27,809 (7.90%)	12,413 (6.68%)	13,453 (8.77%)	1,943 (15.2%)	**<0.001**
Medication use					
NSAIDs	130,850 (37.2%)	60,246 (32.4%)	62,631 (40.8%)	7,973 (62.5%)	**<0.001**
Antiplatelet	2,943 (0.84%)	1,036 (0.56%)	1,506 (0.98%)	401 (3.15%)	**<0.001**
Glucocorticoid	3,692 (1.05%)	1,334 (0.72%)	1,820 (1.19%)	538 (4.22%)	**<0.001**
Vitamin K antagonist	3,384 (0.96%)	1,356 (0.73%)	1,642 (1.07%)	386 (3.03%)	**<0.001**
LMWH	46 (0.01%)	14 (0.01%)	23 (0.01%)	9 (0.07%)	**N/A**
PPIs	30,839 (8.76%)	11,809 (6.35%)	15,744 (10.3%)	3,286 (25.8%)	**<0.001**
Frailty indicators					
Weight loss	53,932 (15.3%)	0 (0.00%)	48,314 (31.5%)	5,618 (44.1%)	**<0.001**
Exhaustion	39,300 (11.2%)	0 (0.00%)	30,984 (20.2%)	8,316 (65.2%)	**<0.001**
Slow gait speed	22,771 (6.47%)	0 (0.00%)	13,752 (8.96%)	9,019 (70.8%)	**<0.001**
Low physical activity	70,428 (20.0%)	0 (0.00%)	60,577 (39.5%)	9,851 (77.3%)	**<0.001**
Low grip strength	44,651 (12.7%)	0 (0.00%)	36,071 (23.5%)	8,580 (67.3%)	**<0.001**

a Displayed as mean (standard deviation).

b Displayed as median (interquartile range, IQR).

Numbers are n (%) unless otherwise stated. Boldface indicates statistical significance (*p < 0.05, **p < 0.01, ***p < 0.001). TDI, Townsend deprivation index; BMI, Body mass index; NSAIDs, Nonsteroidal anti-inflammatory Drugs; LMWH, Low molecular weight heparin; PPIs, Proton pump inhibitors.

### 3.2 Frailty and incident gastrointestinal bleeding risk

During a median follow-up of 14.7 years (interquartile range: 13.9–15.4 years), 20,105 incident GIB events were recorded. Table 2 shows the association between frailty status and GIB risk through multivariable-adjusted analyses. After fully adjusting for covariates, the risk of GIB was significantly higher in both the pre-frail group (HR = 1.15, 95% CI: 1.11–1.18, Table 2) and frail group (HR = 1.53, 95% CI: 1.44–1.62, Table 2) compared to the non-frail group (*P* for trend < 0.001). Furthermore, each 1-point increase in frailty score was associated with a 19% higher risk of bleeding (Supplementary Table S4). Kaplan–Meier survival analysis showed significant divergence in cumulative GIB incidence across frailty categories (log-rank test *p* < 0.001; Supplementary Figure S2).

**Table 2:** Risk of GIB associated with baseline frailty status.

	Non-frail	Pre-frail	Frail	*p* for trend
Overall population (*N* = 352,060)				
No. of participants	**185,889**	**153,424**	**12,747**	**-**
No. of incident GIB	**9,232**	**9,502**	**1,371**	**-**
Follow-up, years Median (IQR)	**14.7 (14.0–15.4)**	**14.6 (13.9–15.3)**	**14.5 (13.7–15.3)**	**-**
Hazard ratio for incident GIB (95% CI, *p* value)				
Unadjusted	Reference	**1.26 (1.22–1.29, *p* < 0.001)**	**2.24 (2.12–2.37, *p* < 0.001)**	**<0.001**
Adjusted model 1	Reference	**1.26 (1.23–1.30, *p* < 0.001)**	**2.20 (2.09–2.34, *p* < 0.001)**	**<0.001**
Adjusted model 2	Reference	**1.20 (1.16–1.23, *p* < 0.001)**	**1.84 (1.73–1.95, *p* < 0.001)**	**<0.001**
Adjusted model 3	Reference	**1.15 (1.11–1.18, *p* < 0.001)**	**1.53 (1.44–1.62, *p* < 0.001)**	**<0.001**

Adjusted model 1: Age and sex were adjusted; Adjusted model 2: Townsend deprivation index, education level, ethnicity, BMI, smoking status and alcohol intake frequency were additionally adjusted; Adjusted model 3: Diabetes, Hypertension, Cardiovascular disease, Chronic kidney failure, Cancer, High-risk digestive diseases, NSAIDs, other high-bleeding-risk medications, PPIs were additionally adjusted; P for trend was calculated by using median frail score value (0, 1 and 3) of each frailty status. Other high-bleeding-risk medications included Antiplatelet, Glucocorticoid, Vitamin K antagonist, LMWH; Boldface indicates statistical significance (*p < 0.05, **p < 0.01, ***p < 0.001). GIB, Gastrointestinal bleeding; IQR, inter-quartile range; CI, confidence interval; BMI, Body mass index; NSAIDs, Nonsteroidal anti-inflammatory Drugs; PPIs, Proton pump inhibitors; LMWH, Low molecular weight heparin.

**Figure 1:**
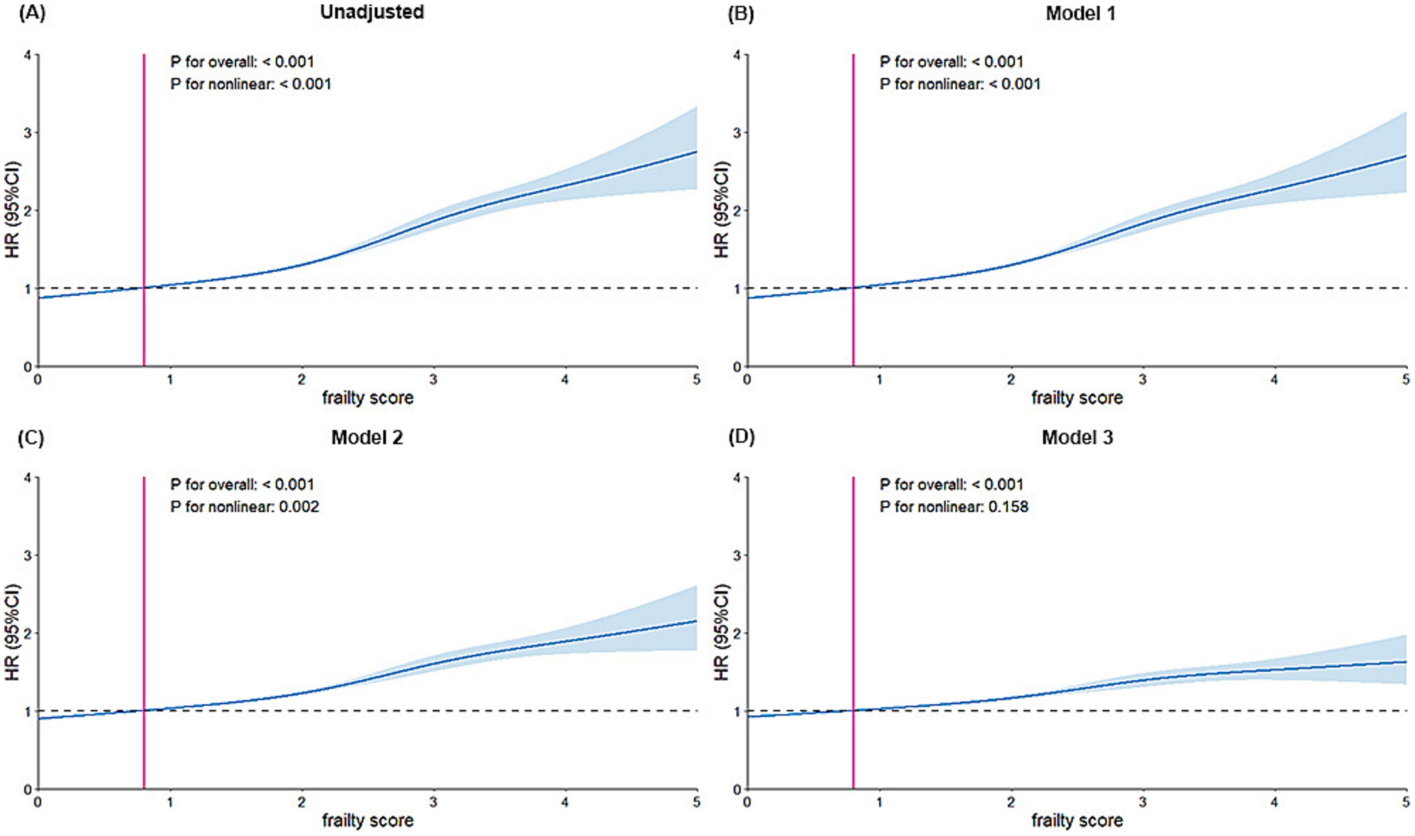
Dose–response association of frailty with the risks of incident GIB. **(A)** Unadjusted model, **(B)** Adjusted model 1: Adjusted for age and sex. **(C)** Adjusted model 2: Adjusted further for Townsend deprivation index, education level, ethnicity, body mass index, smoking status, and alcohol drinking. **(D)** Adjusted model 3: Additionally adjusted for diabetes, hypertension, cardiovascular disease, chronic kidney failure, cancer, high-risk digestive diseases, NSAIDs, other high-bleeding-risk medications and PPIs. Other high-bleeding-risk medications included Antiplatelet, Glucocorticoid, Vitamin K antagonist, LMWH; HR, hazard ratio; CI, confidence interval; GIB, Gastrointestinal bleeding; NSAIDs, Nonsteroidal anti-inflammatory Drugs; PPIs, Proton pump inhibitors; LMWH, Low molecular weight heparin.

As shown in Figure 1, initial analyses of the unadjusted model and the first two adjusted models revealed a nonlinear association between frailty phenotype and GIB risk (*P* for nonlinearity < 0.001), with the risk increasing monotonically across frailty score percentiles. In contrast, this nonlinear relationship was no longer observed in Model 3; however, a statistically significant linear trend persisted (*P* for overall < 0.001).

### 3.3 Additional analysis

**Figure 2:**
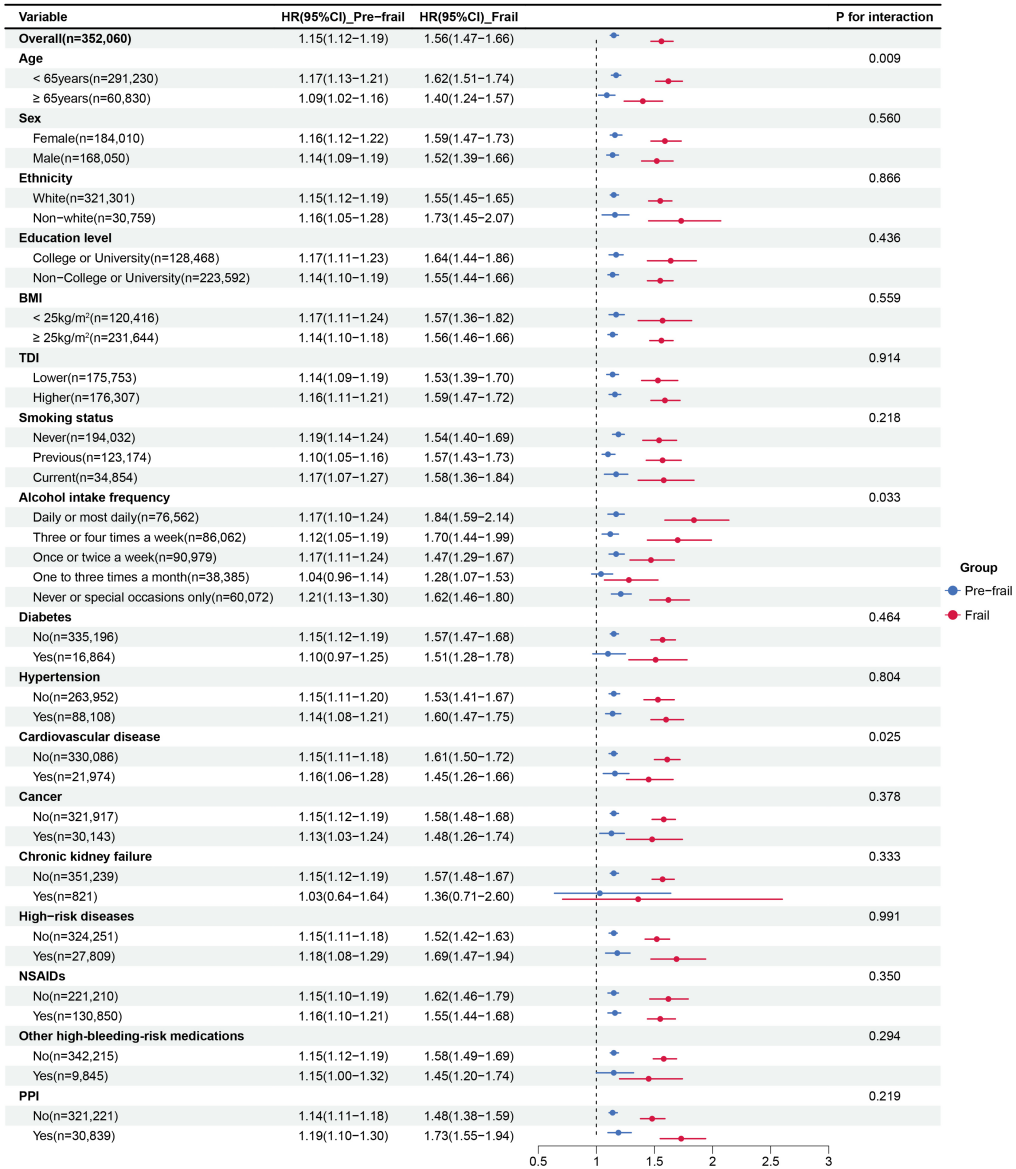
Subgroup analysis for the association between frailty status and GIB risk. Subgroup analyses were conducted for pre-frailty and frailty compared with non-frailty. Multivariable Cox models were adjusted for age, sex, Townsend deprivation index, education level, ethnicity, BMI, smoking status, alcohol intake frequency, diabetes, hypertension, cardiovascular disease, chronic kidney failure, cancer, high-risk digestive diseases, NSAIDs, other high-bleeding-risk medications and PPIs. Other high-bleeding-risk medications included Antiplatelet, Glucocorticoid, Vitamin K antagonist, LMWH; HR, hazard ratio; GIB, Gastrointestinal bleeding; BMI, Body mass index; NSAIDs, Nonsteroidal anti-inflammatory Drugs; PPIs, Proton pump inhibitors; LMWH, Low molecular weight heparin.

Subgroup analysis (Figure 2) revealed that the positive association between frailty and GIB was consistently observed across all populations. In the subgroups based on age and alcohol intake frequency, the relationship between physical frailty and GIB risk was more pronounced in individuals aged ≥65 years (*P* for interaction = 0.009) and those with high-frequency alcohol intake (*P* for interaction = 0.033) compared to others. A history of cardiovascular disease showed an antagonistic effect on the positive association between frailty and GIB events (*P* for interaction = 0.025). No significant interactions were found for other factors (*P* for interactions > 0.05).

The robustness of the study findings was confirmed through multidimensional validation in the sensitivity analysis (details in Supplementary Tables S5–S7). First, after excluding newly diagnosed GIB cases within the first 2 years of follow-up (*N* = 2,611), the hazard ratios remained significant for both the pre-frail (HR = 1.14, 95% CI: 1.10–1.18) and frail groups (HR = 1.49, 95% CI: 1.40–1.60; Supplementary Table S5). Second, after adjusting for the competing risk of all-cause mortality using the Fine & Gray model, the effect estimates remained consistent (pre-frail: HR = 1.15, 95% CI: 1.11–1.18; frail: HR = 1.53, 95% CI: 1.44–1.62; Supplementary Table S6). Third, an independent analysis of the five frailty phenotype components showed that each component was independently associated with GIB risk (all *p* < 0.001), supporting the biological plausibility of the main findings (Supplementary Table S7).

## 4 Discussion

This large prospective cohort study identifies a novel association between frailty and incident gastrointestinal bleeding. Following comprehensive adjustment for demographic characteristics, lifestyle factors, and comorbidities, pre-frail and frail participants exhibited 15 and 53% higher risks of GIB, respectively, compared with non-frail individuals. These associations remained robust across multiple sensitivity analyses. This finding highlights the clinical significance of frailty as a key predictor and potential contributing factor in GIB development. Implementing preventive and intervention strategies targeting frailty may help mitigate the risk of GIB.

This study is the first to systematically investigate the longitudinal relationship between frailty and GIB risk using a population-based prospective cohort design. Previous research has primarily focused on the prognostic implications of frailty in patients with established GIB. Notably, a Canadian prospective cohort study (*N* = 298) demonstrated that assessments using the CSHA-CFS Clinical Frailty Scale served as an independent predictor of in-hospital mortality (adjusted OR = 1.66) and 30-day mortality (adjusted OR = 1.83) in patients with upper gastrointestinal bleeding (17). Another retrospective analysis (*N* = 757,920) revealed that frail GIB patients experienced significantly elevated rates of periprocedural adverse events (49.2% vs. 25.5%) and mortality (4.1% vs. 0.4%) compared to non-frail counterparts (18). While existing studies underscore the importance of frailty management in the secondary prevention of GIB, their methodological limitations—such as retrospective designs and reliance on administrative data for frailty assessment—may compromise the reliability of causal inferences. In contrast, the current study utilizes a clinically validated frailty phenotype (modified Fried criteria), which more accurately reflects biological aging characteristics compared to definitions derived from administrative data. We provide the first evidence that both pre-frailty (HR = 1.15) and frailty (HR = 1.53) serve as independent risk factors for incident GIB. This fundamentally broadens the investigative scope of frailty in GIB, shifting the focus from post-event prognostic evaluation to pre-event risk prediction. This finding addresses a critical knowledge gap regarding the role of frailty in the primary prevention of GIB. In addition, previous research suggests that nutritional and exercise interventions in prefrail populations may reduce morbidity and mortality (22, 23). The identification of pre-frailty as an independent risk factor for GIB highlights this transitional state as a critical window for clinical intervention, representing a novel application of frailty assessment informed by risk prediction findings and complementing its well-established prognostic value.

The pathophysiological mechanisms underlying the association between frailty and gastrointestinal bleeding are not yet fully understood. However, recent research has begun to investigate potential pathways: (a) Neuroendocrine dysregulation and epithelial barrier impairment: Frailty is characterized by a disruption of neuroendocrine homeostasis, particularly dysfunction of the hypothalamic–pituitary–adrenal (HPA) axis and reduced activity of the growth hormone/insulin-like growth factor-1 (GH/IGF-1) axis (24–26). These disruptions have been shown to impair intestinal epithelial cell proliferation and reduce tight junction proteins (27), such as occludin and claudin (26), thereby increasing intestinal permeability and compromising mucosal barrier function. (b) Chronic inflammation and immune dysregulation: Frail individuals exhibit a persistent state of systemic inflammation, partially driven by the senescence-associated secretory phenotype (SASP), which promotes the sustained release of pro-inflammatory cytokines, particularly interleukin-6 (IL-6) and tumor necrosis factor-alpha (TNF-*α*), from monocytes and senescent cells (12, 28, 29). These inflammatory mediators heighten the risk of GIB through two primary mechanisms: TNF-α promotes endothelial cell apoptosis and microvascular remodeling (30), whereas IL-6 impedes ulcer healing (31). (c) Metabolic dysregulation and impaired hemostasis: Metabolic disturbances commonly observed in frailty contribute significantly to gastrointestinal vulnerability and bleeding risk. One key mechanism involves insufficient reserves of branched-chain amino acids (BCAAs), especially leucine, which impairs glutamine synthesis (32). Glutamine plays a vital role in maintaining the integrity and thickness of the intestinal mucus layer (33). Additionally, impaired *γ*-carboxylation of vitamin K-dependent coagulation factors and platelet dysfunction due to zinc deficiency further compromise hemostasis (13, 34, 35). (d) Medication-related mucosal injury: Polypharmacy is prevalent in frail populations and poses additional risks. Especially the combination of NSAIDs and antiplatelet agents, significantly weaken gastric mucosal defenses through COX-1 inhibition (36). Moreover, prolonged proton pump inhibitors (PPIs) use may induce intestinal Enterochromaffin-like cell (ECL) proliferation (37). Despite significant recent advances, evidence remains limited, and further research is required to elucidate the underlying mechanisms.

Our findings have important implications for clinical practice. In this study, the association between frailty and GIB risk was more pronounced in participants aged ≥65 years than in younger individuals. This may be partly explained by the higher prevalence of frailty in older adults, which could contribute to their increased susceptibility to GIB (7, 14, 38). These findings underscore the potential value of frailty screening in guiding targeted preventive strategies for older adults, who may benefit from close monitoring for early signs of GIB, proactive management of comorbidities and medication use, and multidisciplinary interventions, including nutritional support, physical therapy, and geriatric care, to mitigate adverse outcomes (7, 10). Additionally, it is hypothesized that frequent alcohol consumption may amplify the association between frailty and GIB. The direct damaging effects of alcohol on the gastrointestinal mucosa, along with its interference in coagulation processes (39), may make frail individuals more vulnerable to bleeding events. Therefore, quantitative control of alcohol intake could serve as a viable strategy for mitigating bleeding risk in this population. It is noteworthy that individuals without a history of cardiovascular disease may exhibit an increased risk of bleeding. This may be due to the absence of routine gastrointestinal protective measures in this subgroup, as they are typically not prescribed anticoagulation therapy (40). Therefore, clinicians should exercise increased vigilance regarding bleeding risk in this patient population.

Given the limited number of longitudinal studies on the association between frailty and GIB, this study provides the first prospective evidence in this area. The large sample size, extended follow-up period, and application of multivariable statistical analyses enhance the credibility of the findings. However, several limitations should be acknowledged. First, the observational cohort design does not allow for a definitive causal inference between frailty status and GIB. Future studies employing Mendelian randomization may help clarify causality. Second, most frailty indicators (including weight loss, exhaustion, slow walking speed) were based on self-reported data (11), which may introduce recall or social desirability bias. Additionally, the Fried frailty phenotype does not capture other important dimensions of frailty, such as cognitive, social, and psychological components, all of which may influence GIB risk. Future research should consider incorporating objective measures and multidimensional frailty assessment tools, as the predictive utility of the frailty index has been well established in numerous studies (15, 41). Third, although multiple covariates were included, residual confounding may still influence the occurrence of gastrointestinal bleeding. Future studies should consider incorporating additional factors known to affect bleeding risk, such as dietary habits and *Helicobacter pylori* infection status (2). Fourth, the current study did not investigate changes in frailty status over time or their potential impact on GIB risk (41). Finally, as the study population primarily consisted of UK residents of predominantly white ethnicity, the generalizability of the results may be limited and should be validated in more diverse cohorts.

The findings of this study offer an original contribution to aging research and public health. In the context of global demographic shifts, frailty represents a modifiable indicator that extends beyond conventional risk factors in identifying older adults at heightened risk of GIB (7). The integration of validated frailty assessments into community health surveillance and routine geriatric care may facilitate the early identification of high-risk individuals, thereby informing targeted preventive strategies (10). These findings provide a scientific basis for the development and implementation of interventions aimed at reducing the incidence of serious GIB events and alleviating the associated healthcare burden in aging populations. Importantly, by advancing the understanding of GIB etiology, this study supports evidence-based approaches to addressing the challenges of population aging and promotes the overarching goal of healthy aging within public health systems.

This study demonstrates that frailty significantly increases the risk of gastrointestinal bleeding, with the risk rising proportionally with the severity of frailty. Early identification of at-risk individuals is essential for the prevention of GIB. Comprehensive, targeted interventions for frail patients are crucial to improving overall health and reducing the risk of GIB.

## Data Availability

The data analyzed in this study is subject to the following licenses/restrictions: this study has been conducted using the UK Biobank Resource (www.ukbiobank.ac.uk). The UK Biobank will provide its source data to qualified researchers for health-related research in the public interest, with no exclusive or preferential access granted to any party. All researchers must follow UK Biobank’s standard application process and approval criteria. Requests to access these datasets should be directed to www.ukbiobank.ac.uk.
