# Supplementary material for: Frailty and risk of gastrointestinal bleeding: a prospective cohort study based on UK biobank
Source: Front Public Health. 2025 Jul 4;13:1625869. doi: 10.3389/fpubh.2025.1625869 (PMC12270847; doi:10.3389/fpubh.2025.1625869)
Supplement: Supplementary file 1 [file Supplementary_file_1.docx]

**Supplemental Material**

**Frailty and risk of gastrointestinal bleeding: a prospective cohort study based on UK biobank**

**Chenao Zhang^1,2#^, Qiming Huang^1#^, Xingyu Liu^1^，Jiren Wang^1^，Junyan Wang^1^, Jian Song^2^, Rong Song^2^, Hong Su^2*^，Qiao Mei^1*^**

**Authors:**

Chenao Zhang, Department of Gastroenterology, The First Affiliated Hospital of Anhui Medical University, Jixi Road 218, Hefei, 230022, China. E-mail address: 972456907@qq.com.

Qiming Huang, Department of Gastroenterology, The First Affiliated Hospital of Anhui Medical University, Jixi Road 218, Hefei, 230022, China. E-mail address: qimhuang@163.com

Xingyu Liu, Department of Gastroenterology, The First Affiliated Hospital of Anhui Medical University, Jixi Road 218, Hefei, 230022, China. E-mail address: lxyuuu0807@163.com.

Jiren Wang, Department of Gastroenterology, The First Affiliated Hospital of Anhui Medical University, Jixi Road 218, Hefei, 230022, China. E-mail address: [985378556@qq.com](mailto:985378556@qq.com).

Junyan Wang, Department of Gastroenterology, The First Affiliated Hospital of Anhui Medical University, Jixi Road 218, Hefei, 230022, China. E-mail address: 17718170143@163.com.

Jian Song, Department of Epidemiology and Health Statistics, School of Public Health, Anhui Medical University, 81 Meishan Road, Hefei, Anhui Province, 230032, China. E-mail address: [sj@ahmu.edu.cn](mailto:sj@ahmu.edu.cn).

Rong Song, Department of Epidemiology and Health Statistics, School of Public Health, Anhui Medical University, 81 Meishan Road, Hefei, Anhui Province, 230032, China. E-mail address: [1623555029@qq.com](mailto:1623555029@qq.com).

**Correspondences:**

Hong Su, Department of Epidemiology and Health Statistics, School of Public Health, Anhui Medical University, 81 Meishan Road, Hefei, Anhui Province, 230032, China. E-mail address: suhong5151@sina.com.

Qiao Mei, Department of Gastroenterology, The First Affiliated Hospital of Anhui Medical University, Jixi Road 218, Hefei, 230022, China. E-mail address: [meiqiaomq@aliyun.com](mailto:meiqiaomq@aliyun.com).

**Supplementary Figures and Tables**

**Supplementary Figure S1.** Flowchart of participants in current study.

**Supplementary Figure S2.** Cumulative hazard curve for gastrointestinal bleeding (GIB).

**Supplementary Table S1.** Definition of frailty phenotype and cut-off points in UK Biobank.

**Supplementary Table S2.** Outcome Definitions in UK Biobank Study

**Supplementary Table S3.** Subgroups and data codes of variables in the UK Biobank.

**Supplementary Table S4.** Risk of GIB associated with baseline frailty score.

**Supplementary Table S5.** Risk of GIB associated with frailty status after removing the participants with GIB within 2 years.

**Supplementary Table S6.** Association of Frailty and GIB Using Fine & Gray Models for Competing Risk.

**Supplementary Table S7.** Individual Components of Frailty and Their Association With Incident GIB.

Figure S1: Flowchart of participants in current study.


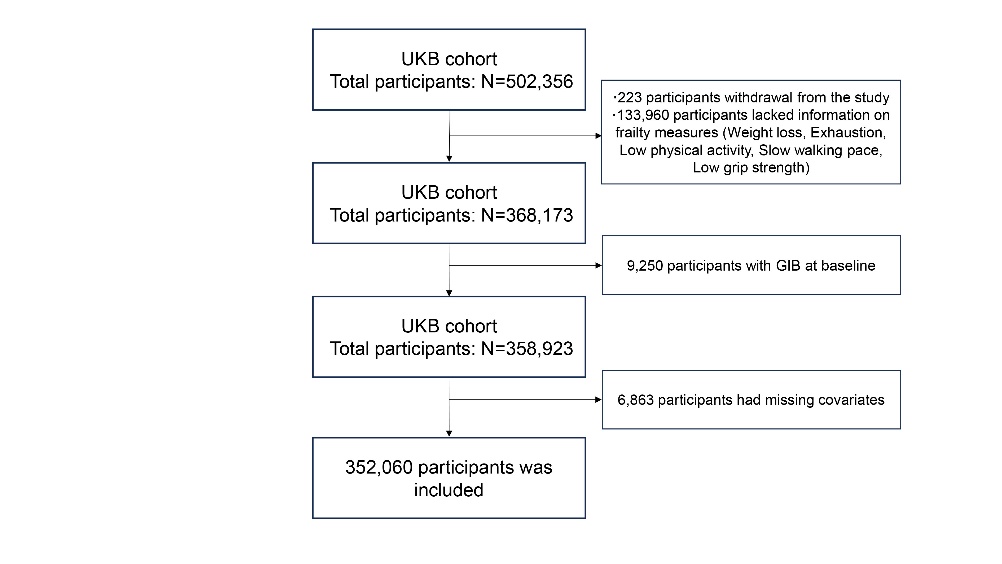


Abbreviations: UKB: UK Biobank; GIB: Gastrointestinal bleeding.

Figure S2: Cumulative risks of incident Gastrointestinal bleeding in accordance with frailty profile.


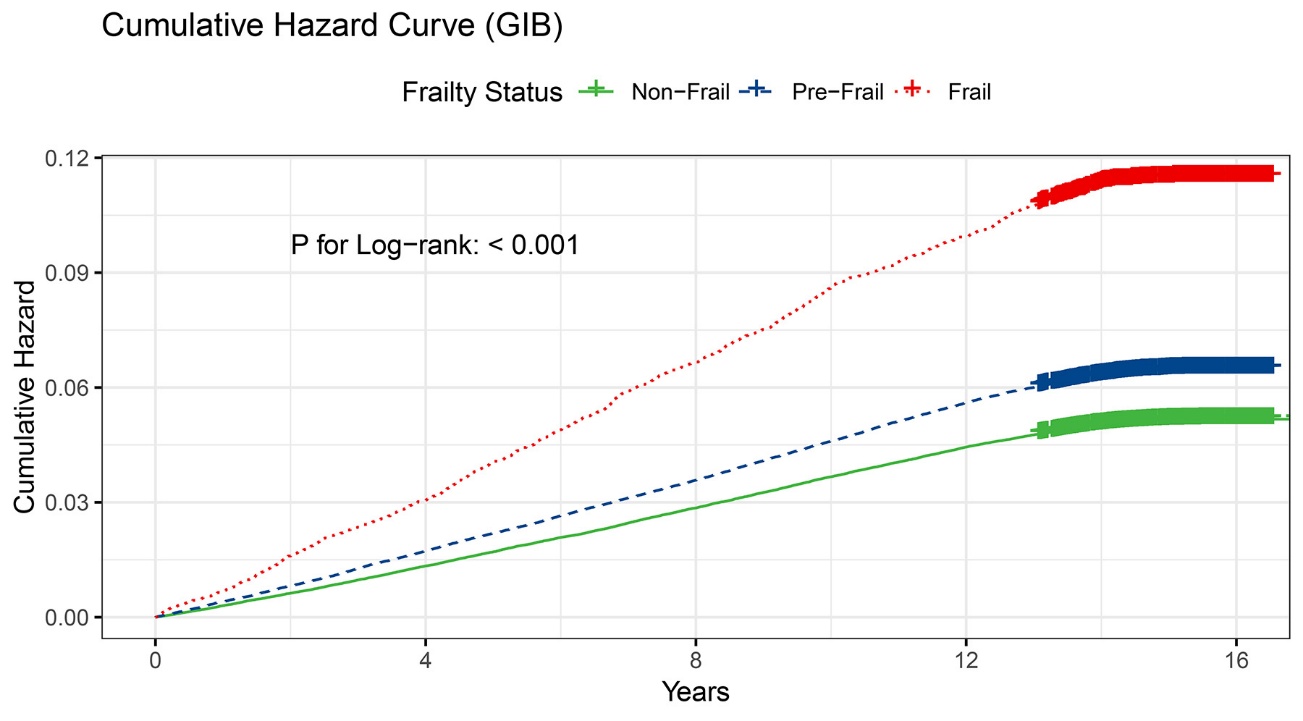


Abbreviations: GIB: Gastrointestinal bleeding.

**Supplementary Table S1:** Frailty Definition and Cut-off Points in UK Biobank Study

| **Individual frailty phenotype components** | **Criteria** | **Field IDs** |
| --- | --- | --- |
| Weight loss | Self-reported: “Compared with one year ago, has your weight changed?”  Response: yes, lost weight = 1; other = 0;  Do not know/Prefer not to answer = missing data. | 2306 |
| Exhaustion | Self-reported: “Over the past two weeks, how often have you felt tired or had little energy?”  Response: more than half the days or nearly every day = 1; other = 0;  Do not know/Prefer not to answer = missing data. | 2080 |
| Slow gait speed | Self-reported: “How would you describe your usual walking pace?”  Response: slow = 1; other = 0;  Do not know/Prefer not to answer = missing data. | 924 |
| Low grip strength | Measured grip strength expressed in kg by sex- and BMI- adjusted cut-off points. Cut-off points:  Men  If BMI ≤24.0 kg/m^2^ & grip strength ≤29 kg  If BMI 24.1 to 26.0 kg/m^2^ & grip strength ≤30 kg  If BMI 26.1 to 28.0 kg/m^2^ & grip strength ≤30 kg  If BMI >28.0 kg/m^2^ & grip strength ≤32 kg   Women  If BMI ≤23.0 kg/m^2^ & grip strength ≤17 kg  If BMI 23.1 to 26.0 kg/m^2^ & grip strength ≤17.3 kg If BMI 26.1 to 29.0 kg/m^2^ & grip strength ≤18 kg  If BMI >29.0 kg/m^2^ & grip strength ≤21 kg  If data on BMI or grip strength is not available = missing data. | 31, 21001, 46, 47 |
| Low physical activity | Quintiles of sex- and age-specific levels of total MET minutes per week derived from IPAQ.  The lowest 20% of total MET minutes per week = 1; other = 0;  No response/ Prefer not to answer = missing data. | 31, 21022, 22037, 22038, 22039 |

Abbreviations: BMI, body mass index; MET, metabolic equivalent; IPAQ, International Physical Activity Questionnaire.

**Supplementary Table S2:** Outcome Definitions in UK Biobank Study

| **ICD-9** | **ICD-10** | **Death register** | **Self-reported** |
| --- | --- | --- | --- |
| **5307、5310、5314、5320、5324、5326、5334、5344、5693、5780、5781、5789、4560** | **K25.0、K25.2、K25.4、K25.6、K26.0、K26.2、K26.4、K26.6、K27.0、K27.2、K27.4、K27.6，K28.0、K28.2、K28.4、K28.6、K92.0、K92.1、K92.2、K62.5、I85.0、 I98.3** | **K22.6、K25.0、K25.4、K25.6、K26.4、K26.6、K27.0、K27.4、K28.4、K29.0、K62.5、K92.0、K92.2、I85.0** | **20002（1191）** |

Note: Variable definitions constructed using ICD-9, ICD-10 codes, death register and self-reported data fields with choice-, disease- or procedure-specific codes between brackets are shown.

Abbreviations: ICD, International Classification of Diseases.

**Supplementary Table S3:** Subgroups and data codes of variables in the UK Biobank.

| Covariates | Subgroups | codes |
| --- | --- | --- |
| Age | - | 21022 |
| Sex | Male/Female | 31 |
| Ethnicity | White/Non-white | 21000 |
| Townsend deprivation index | - | 22189 |
| Smoking status | Never/Previous/Current | 20116 |
| Alcohol intake frequency | Daily or most daily/ Three or four times a week/ Once or twice a week/ One to three times a month/ Never or special occasions only | 1558 |
| Body mass index | - | 21001 |
| Education | College or University/Non College or University | 6138 |
| Hypertension | - | 20002（1065、1072）  6150 |
| Diabetes | - | 2443、20002(1220、1221、1222、1223、1276、1468、1607) |
| Cardiovascular disease | heart/cardiac problem, angina, heart attack/myocardial infarction, heart failure/pulmonary odema, cardiomyopathy, stroke, transient ischaemic attack (tia), subarachnoid haemorrhage, atrial fibrillation, brain haemorrhage, ischaemic stroke, hypertrophic cardiomyopathy (hcm / hocm) | 20002（1066、1074、1075、1076、1079、1081、1082、1086、1471、1491、1583、1588） |
| Chronic kidney disease | renal/kidney failure, Renal failure requiring dialysis, renal failure not requiring dialysis, polycystic kidney, kidney nephropathy, Iga nephropathy, diabetic nephropathy | 20002（1192、1193、1194、1427、1519、1520、1607） |
| High risk digestive diseases | oesophageal disorder, stomach disorder, gastro-oesophageal reflux (gord) / gastric reflux, oesophagitis/barretts oesophagus, oesophageal varicies, gastric/stomach ulcers, gastritis/gastric erosions, liver failure/cirrhosis, peptic ulcer, duodenal ulcer, diverticular disease/diverticulitis, colitis/not crohns or ulcerative colitis, rectal or colon adenoma/polyps, inflammatory bowel disease, crohns disease, ulcerative colitis, primary biliary cirrhosis, alcoholic liver disease / alcoholic cirrhosis, anal fissure, haemorrhoids / piles, liver/biliary/pancreas problem | 1134、1135、1138、1139、1141、1142、1143、1158、1400、1457、1458、1459、1460、1461、1462、1463、1506、1604、1504、1505、1136 |
| Cancer | - | 134、2453 |
| Nonsteroidal anti-inflammatory Drugs | Aspirin, Ibuprofen, Aceclofenac, Acemetacin, Azapropazone, Bufexamac, Celecoxib, Choline magnesium Trisalicylate, Desketoprofen, Diclofenac, Diflunisal, Etodolac, Felbinac, Fenoprofen, Flurbiprofen, Hydroxyphenylbutazone, Indometacin, Ketoprofen, Ketorolac, Lornaxicam, Magnesium salicylate, Mefenamic acid, Meloxicam, Nabumetone, Naproxen, Oxyphenbutazone, Phenylbutazone, Piroxicam, Salsalate, Sulindac, Tenoxicam, Tiaprofenic acid, Tolfenamic acid, Tolmetin, paracetamol, Celecoxib, Etoricoxib, Parecoxib, Rofecoxib, Valdecoxib | 6154, 1140909772, 1140861804, 1140868226, 1140861806, 1140882392, 1140882268, 1140882108, 1140882190, 1140868282, 1140872040, 1141163138, 1141167848, 1140909888, 1140871080, 1140925942, 1140923344, 1140856336, 1141167844, 1140861808, 1140882192, 1141151924, 1140868264, 1140882106, 1140856394, 1141164050, 1141164044, 1140856314, 1140863514, 1140856220, 1141177826, 1140872032, 1140864860, 1140856212, 1141188536, 1140917408, 1140868294, 1140856440, 1140856214, 1140856344, 1140928656, 1141153082, 1141153134, 1140871370, 1140871374, 1141191742, 1140871388, 1141190952, 1140871386, 1140871394, 1141182868, 1141181112, 1141165574, 1140877866, 1140853090, 1140871408, 1140853094, 1141169472, 1140871310, 1141157412, 1140925868, 1140871392, 1140871404, 1141165754, 1140871396, 1141181752, 1140877864, 1140871406, 1141187776, 1141182642, 1140877862, 1140853100, 1140877854, 1140910496, 1140877858, 1140871416, 1140925806, 1140875278, 1141163014, 1140871514, 1140871492, 1140871556, 1140871344, 1140871100, 1141182010, 1140855134, 1140853022, 1140871662, 1140853066, 1140877878, 1140871606, 1141176886, 1140928844, 1141163126, 1141181872, 1140856338, 1141164746, 1141162950, 1140884488, 1140921828, 1140917394, 1141146404, 1141157112, 1140871260, 1141153330, 1140871282, 1141145626, 1140871094, 1140882264, 1140871284, 1140877876, 1140871450, 1141173776, 1141193170, 1141176878, 1141191028, 1140875282, 1140871188, 1140884498, 1140871672, 1141182674, 1140871202, 1140871218, 1140917074, 1140871226, 1140871228, 1140877880, 1141152674, 1140871590, 1140871276, 1141170824, 1140871360, 1141146400, 1141156966, 1140871236, 1140871238, 1140910686, 1140871348, 1140871354, 1140853108, 1140853110, 1140871442, 1140909936, 1141181656, 1140871336, 1141157452, 1140871434, 1141146568, 1140871274, 1141150922, 1141164750, 1141169802, 1140916866, 1140871532, 1140871506, 1140884558, 1140927756, 1140871528, 1140917406, 1141168652, 1140888872, 1140871468, 1140871206, 1140871196, 1140921968, 1141184156, 1140871542, 1140916806, 1140926732, 1140926792, 1140926796, 1141168702, 1140926794, 1140853012, 1140909354, 1140875336, 1140911560, 1140871472, 1140871462, 1140871568, 1140922318, 1140871516, 1140871522, 1140875680, 1141153346, 1140871654, 1140871666, 1141169526, 1141182754, 1140871582, 1140871546, 1140871490, 1140925808, 1140850818, 1140875338, 1140911098, 1141167426, 1140871102, 1140871180, 1140871430, 1140871564, 1140871092, 1140853018, 1141145722, 1141170278, 1141182704, 1140871604, 1140871616, 1140868336, 1140875346, 1140871614, 1141199380, 1141183944, 1140864412, 1140875270, 1140853030, 1140928840, 1140875268, 1140881118, 1140853068, 1141190644, 1140871256, 1140871484, 1140871248, 1140923920, 1140877872, 1140877868, 1140877874, 1140871174, 1140871168, 1141184162, 1140888772, 1140926016, 1140928954, 1141156856, 1141163764, 1141169026, 1141185058, 1141185060, 1141188500, 1141188516, 1141188780, 1141188784, 1141190956, 2038460150, 1140868240, 1140868274, 1140868278, 1140868322, 1140871968, 1140872044, 1140882266, 1140882394, 1140884408, 1140928946, 1140928956, 1141168646, 1141183332, 1140856238, 1140856240, 1140856242, 1140868304, 1140882262, 1140868312, 1141168560, 1141168562, 1141176668, 1141176670, 1141176662, 1141180076, 1141180078, 1141180126, 1141165244, 1141192798, 1141165252, 1141165312, 1141165254, 1141165314, 1141184290, 1141184292 |
| Antiplatelet | Clopidogrel, Dipyridamole, Ticlopidine | 1140861776, 1141167848, 1140861790, 1141168318, 1140861778, 1141167844, 1140911710, 1140861780, 1141168322, 1141163328, 1141163324, 1140851930 |
| Vitamin K antagonist | Acenocoumarol, Phenindione, Warfarin | 1140909770, 1140861704, 1141164760, 1140861696, 1140864122, 1140861702, 1140861698, 1140910832, 1140888266 |
| Low molecular weight heparin | Bemiparin, Certoparin, Dalteparin, Enoxaparin, Reviparin, Tinzaparin | 1140926360, 1141189054, 1140926444, 1140861594, 1141171374, 1140888204, 1140861588, 1140861584, 1140861602, 1141171364, 1140888206, 1141189212, 1141189210 |
| Glucocorticoid | Prednisone, Prednisolone, Methylprednisolone, Dexamethasone, Cortisone, Triamcinolone, Betamethasone, Hydrocortisone, Fludrocortisone, desoxymethasone | 1140868364, 1140874930, 1141157402, 1140874976, 1140854700, 1140874816, 1140857534, 1141173346, 1140868426, 1140868370, 1140874976, 1140857672, 1140857678, 1140874790, 1140874896, 1140884672, 1140884704, 1140910424, 1140910634, 1141157294, 1140910484, 1140874936 |
| Proton pump inhibitors | Esomeprazole, Lansoprazole, Omeprazole, Pantoprazole, Rabeprazole | 1140929012, 1141184174, 1141180462, 1141168822, 1141177526, 1141168824, 1141184176, 1140864752, 1140909578, 1141177532, 1140865634, 1141187060, 1141168590, 1141164616, 1141168584, 1140850960, 1141190552, 1140923688 |

**Supplementary Table S4:** Risk of GIB associated with baseline frailty score.

| Frailty score |  | HR (95% CI) | P value |
| --- | --- | --- | --- |
| Overall population (N=352,060) | | | |
| Unadjusted | Per 1 score change | 1.25 (1.24, 1.27) | <0.001 |
| Adjusted model 1 | Per 1 score change | 1.36 (1.33, 1.40) | <0·001 |
| Adjusted model 2 | Per 1 score change | 1.27 (1.24, 1.30) | <0·001 |
| Adjusted model 3 | Per 1 score change | 1.19 (1.16, 1.22) | <0·001 |

Note: Adjusted model 1: Age and sex were adjusted; Adjusted model 2: Townsend deprivation index, education level, ethnicity, BMI, smoking status and alcohol drinking were additionally adjusted; Adjusted model 3: Diabetes, Hypertension, Cardiovascular disease, Chronic kidney failure, Cancer, High-risk digestive diseases, NSAIDs, Non NSAIDs, PPIs were additionally adjusted.

Non-NSAIDs included Antiplatelet，Glucocorticoid，Vitamin K antagonist，LMWH;

Abbreviations: GIB: Gastrointestinal bleeding. BMI, body mass index; NSAIDs: nonsteroidal anti-inflammatory drugs; PPIs: Proton pump inhibitors; LMWH: Low molecular weight heparin.

**Supplementary Table S5:** Risk of GIB associated with frailty status after removing the participants with GIB within 2 years.

|  | Non-frail | Pre-frail | Frail | P for trend |
| --- | --- | --- | --- | --- |
| Overall population (N = 349,449) |  |  |  |  |
| No. of participants | 184,726 | 152,179 | 12,544 | - |
| No. of incident GIB | 8,069 | 8,257 | 1,168 | - |
| Follow-up, years  Median (IQR) | 14.7 (14.0-15.4) | 14.6 (13.9-15.3) | 14.5 (13.7-15.3) | - |
| Hazard ratio for incident GIB (95% CI, P value) |  |  |  |  |
| Unadjusted | Reference | 1.25 (1.21-1.29, P < 0.001) | 2.19 (2.06-2.33, P < 0.001) | <0.001 |
| Adjusted model 1 | Reference | 1.26 (1.22-1.29, P < 0.001) | 2.16 (2.03-2.30, P < 0.001) | <0.001 |
| Adjusted model 2 | Reference | 1.19 (1.15-1.23, P < 0.001) | 1.79 (1.67-1.90, P < 0.001) | <0.001 |
| Adjusted model 3 | Reference | 1.14 (1.10-1.18, P < 0.001) | 1.49 (1.40-1.60, P < 0.001) | <0.001 |

Note: Adjusted model 1: Age and sex were adjusted; Adjusted model 2: Townsend deprivation index, education level, ethnicity, BMI, smoking status and alcohol drinking were additionally adjusted; Adjusted model 3: Diabetes, Hypertension, Cardiovascular disease, Chronic kidney failure, Cancer, High-risk digestive diseases, NSAIDs, other high-bleeding-risk medications, PPIs were additionally adjusted; P for trend was calculated by using median frail score value (0, 1 and 3) of each frailty status.

other high-bleeding-risk medications included Antiplatelet，Glucocorticoid，Vitamin K antagonist，LMWH;

Abbreviations: GIB: Gastrointestinal bleeding; IQR: inter-quartile range; BMI, body mass index; NSAIDs: nonsteroidal anti-inflammatory drugs; PPIs: Proton pump inhibitors; LMWH: Low molecular weight heparin.

**Supplementary Table S6:** Association of Frailty and GIB Using Fine & Gray Models for Competing Risk.

| Frailty phenotypes | Non-frailty | Pre-frailty | Frailty |
| --- | --- | --- | --- |
| Hazard ratio (95% CI) | 1.00 (ref.) | 1.15 (1.11-1.18) | 1.53 (1.44-1.62) |

Note: Model adjusted for age(continuous), sex(male/female), Townsend deprivation index(continuous), education level(College or University /Non College or University), ethnicity(White/Non-White), BMI(continuous), smoking status(Never/Previous/Current), alcohol drinking(Daily or most daily/ Three or four times a week/ Once or twice a week/ One to three times a month/ Never or special occasions only), Diabetes(Yes/No), Hypertension(Yes/No), Cardiovascular disease(Yes/No), Chronic kidney failure(Yes/No), Cancer(Yes/No), High-risk digestive diseases(Yes/No), NSAIDs(Yes/No), other high-bleeding-risk medications (Yes/No), PPIs(Yes/No).

Abbreviations: BMI, body mass index; NSAIDs: nonsteroidal anti-inflammatory drugs; PPIs: Proton pump inhibitors.

**Supplementary Table S7:** Individual Components of Frailty and Their Association With Incident GIB.

Note: Adjusted model 1: Age and sex were adjusted; Adjusted model 2: Townsend deprivation index, education level, ethnicity, BMI, smoking status and alcohol drinking were additionally adjusted; Adjusted model 3: Diabetes, Hypertension, Cardiovascular disease, Chronic kidney failure, Cancer, High-risk digestive diseases, NSAIDs, other high-bleeding-risk medications, PPIs were additionally adjusted.

other high-bleeding-risk medications included Antiplatelet，Glucocorticoid，Vitamin K antagonist，LMWH.

Abbreviations: BMI, body mass index; NSAIDs: nonsteroidal anti-inflammatory drugs; PPIs: Proton pump inhibitors; LMWH: Low molecular weight heparin.

| Frailty Component | Unadjusted (HR, 95% CI) | Model 1 (HR, 95% CI) | Model 2 (HR, 95% CI) | Model 3 (HR, 95% CI) |
| --- | --- | --- | --- | --- |
| Weight loss | 1.12 (1.08-1.16) | 1.14 (1.09-1.18) | 1.10 (1.06-1.14) | 1.06 (1.02-1.10) |
| Exhaustion | 1.51 (1.46-1.57) | 1.62 (1.56-1.68) | 1.46 (1.41-1.52) | 1.34 (1.29-1.39) |
| Low physical activity | 1.15 (1.11-1.19) | 1.17 (1.13-1.21) | 1.12 (1.09-1.16) | 1.09 (1.05-1.12) |
| Slow gait speed | 1.89(1.81-1.97) | 1.79 (1.71-1.87) | 1.51 (1.44-1.58) | 1.30 (1.24-1.36) |
| Low grip strength | 1.43(1.37-1.48) | 1.34 (1.29-1.39) | 1.22 (1.17-1.27) | 1.15 (1.10-1.19) |
